# Analyzing soluble and lung smooth muscle laminin isoform expression in guinea pigs exhibiting various allergic response phenotypes

**DOI:** 10.3389/fimmu.2026.1774996

**Published:** 2026-03-24

**Authors:** Ivonne Pacheco-Alba, Angélica Flores-Flores, Blanca Bazán-Perkins

**Affiliations:** 1 Laboratorio de Inmunofarmacología, Instituto Nacional de Enfermedades Respiratorias Ismael Cosío Villegas, Mexico City, Mexico; 2 Tecnológico de Monterrey, Escuela de Medicina y Ciencias de la Salud, Mexico City, Mexico

**Keywords:** bronchoalveolar lavage, guinea pig, laminin isoforms, serum, smooth muscle

## Abstract

**Introduction:**

Ovalbumin (OVA) sensitization in guinea pigs induces a distinct phenotype of allergic responses, among which the asthma model is the most extensively studied. A functionally opposite phenotype is the non-responder (NR), which, despite exhibiting high levels of OVA-specific IgE and IgG1, does not develop airway obstruction or hyperresponsiveness after chronic antigen challenge. This phenotype differs from the asthma model by high expression of the laminin isoform (LN) β2 in pulmonary smooth muscles. However, it remains unknown whether OVA sensitization induces changes in the expression of other LNs. Therefore, the aim of this study was to compare LN expression in guinea pigs with allergic asthma and NR phenotypes and correlate these patterns with distinct pathophysiological responses.

**Methods:**

Guinea pigs were sensitized and challenged with OVA and divided into two groups (asthma model and NR, *n* = 6 per group) according to their response. A control group (*n* = 6) was sensitized and challenged with saline. After 12 antigen challenges at 10-day intervals, differential cell counts were performed in bronchoalveolar lavage (BAL) samples. LN expression in airway smooth muscle (ASM) and intrapulmonary vascular smooth muscle (IVSM) was evaluated by immunohistochemistry. LN presence in BAL fluid and serum was determined by ELISA.

**Results:**

LN α1 and LN α2 expression increased in ASM and IVSM in the asthma model. In contrast, LN β3 increased in ASM and IVSM of NR. LN γ1 increased in IVSM of both phenotypes, whereas LN γ2 remained unchanged. ASM expression of LN α1 and LN α2 correlated with antigen-induced airway obstruction and hyperresponsiveness. Serum LN α1 increased in the asthma model, whereas LN β2 was elevated in NR and, to a lesser extent, in asthma. LN β3 levels increased in serum and BAL in both phenotypes and correlated with inflammatory cell counts.

**Discussion:**

Soluble serum LNs may serve as markers to differentiate antigen-induced phenotypes. LN α1 and LN α2 may contribute to ASM contraction, while LN β3 appears to play a role in antigen-induced airway inflammation.

## 1 Introduction

Lung allergy is an inflammatory condition with variable respiratory symptoms, with airway obstruction being one of its most important features. The degree of obstruction varies according to each individual’s physiological response (1–4), with some individuals remaining asymptomatic despite underlying inflammation (5, 6). In chronic conditions, inflammation can also lead to alterations in the type and amount of proteins present in the basement membrane (BM) (7, 8). These alterations are associated with changes in airway obstruction or loss of lung function, as BM composition can modify the behavior of the surrounding tissues, including smooth muscle (9, 10).

Among the most abundant components of the BM are laminins, which are heterotrimeric glycoproteins composed of one α subunit (with five known isoforms), one β subunit (with three isoforms), and one γ subunit (with three isoforms) (11, 12). Laminins are frequently altered in chronic lung diseases such as chronic obstructive pulmonary disease (COPD) and asthma (13–15). The accumulation of laminin or laminin isoforms (LNs) in airway smooth muscle (ASM) has been linked to changes in muscle function due to the impact of laminin on the mechanical properties of the tissue (15–17). Moreover, the detection of LNs in the bronchoalveolar lavage (BAL) of patients experiencing asthma exacerbations serves as an indicator of BM alterations (18).

Similar to the variability observed in humans, animal models of lung allergy show diverse responses (4, 5, 19–22). Our research group has identified three distinct physiological response types, or phenotypes, in ovalbumin (OVA)-sensitized guinea pig model of lung allergy. The first type, known as responders (R), consistently shows airway obstruction following antigenic challenge, serving as a typical asthma model. The second type, known as variable responders (VR), is similar to R but shows airway obstruction and hyperreactivity only sometimes. The third type, known as non-responders (NR), never shows airway obstruction following antigenic challenge and is hyperreactive in acute models (three OVA challenges) but not in chronic models (more than nine OVA challenges). All these phenotypes exhibit elevated levels of immunoglobulin (Ig) G and OVA-specific IgE. However, NR shows lower levels of IL-4 compared to R and VR but has higher levels of interferon-γ (22, 23). Recently, it has been observed that chronic NR guinea pigs show high expression of LN β2 in ASM and intrapulmonary vascular smooth muscle (IVSM). Particularly, the degree of expression of LN β2 in ASM correlates with reduced antigen-induced airway obstruction and reactivity to histamine, suggesting a potential role of LN β2 in containing obstructive responses (24).

Since R and NR represent sensitized chronic models with distinct physiological responses, this article aims to determine whether the observed differences are related to changes in the type or quantity of LNs expressed in lung smooth muscle. These potential alterations could influence tissue behavior, providing novel possible roles of LNs. We also aim to determine if these LNs are restricted to the tissue or can be found in soluble form in serum or BAL. The analysis of LNs focused on structures such as bronchi and bronchioles, as these tissues are relevant in asthma obstruction (25), and recent findings have shown that the smooth muscle of the trachea does not exhibit protein expression patterns similar to intrapulmonary ASM (26).

## 2 Materials and methods

### 2.1 Animals

Male guinea pigs (strain HsdPoc: DH) with a mean body weight of 300–400 g were used. Animals were kept at the institutional laboratory animal facilities under standard conditions: temperature 21 °C ± 1 °C, filtered air, 12-h light–dark cycle, and 50%–70% humidity. Throughout the experimental period, the animals were provided unrestricted access to a sterilized pellet diet (2040 Harlan Teklad Guinea Pig Diet, Wisconsin, USA) and water. These housing conditions and subsequent handling procedures were conducted in accordance with the protocols approved by the Scientific and Bioethics Committee of the Instituto Nacional de Enfermedades Respiratorias (IRB organization code: IORG0003948).

### 2.2 Chronic ovalbumin-induced allergic lung inflammation model in guinea pigs

**Figure 1:**
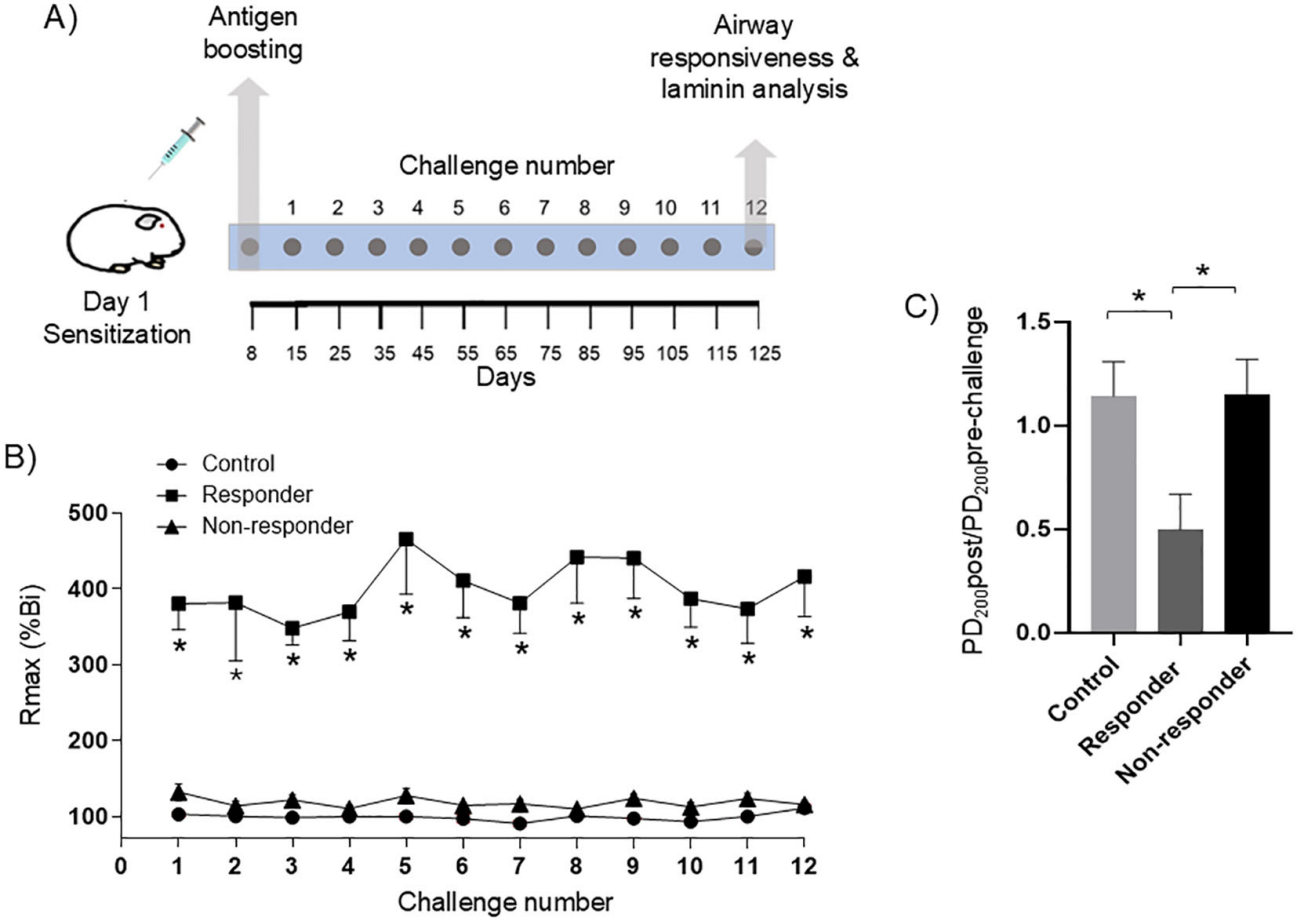
Lung allergy model and broncho-obstructive responses. **(A)** To establish a chronic lung allergy model, guinea pigs were sensitized on day 1 and subsequently boosted on day 8 with the antigen (ovalbumin, OVA). From day 8, animals were subjected to repeated antigenic challenges every 10 days until day 125, for a total of 12 antigen exposures. **(B)** Maximal broncho-obstructive response (Rmax) achieved after antigenic challenge in sensitized guinea pigs. The data represent the percentage of the maximum broncho-obstructive index (Bi) response observed after antigen exposure, compared to the baseline Bi values for each animal. Responder animals achieve an increase of ≥200% in baseline Bi (statistical significance was determined using two-way ANOVA followed by Tukey’s *post hoc* test; * *P* < 0.05). **(C)** Airway responsiveness assessment. Airway responsiveness was assessed on day 125. The ratio between the provocative doses required to induce a ≥200% (PD200) increase in the basal Bi after and before the antigen challenge was compared (PD200post/PD200pre challenge ratio). This ratio was used to quantitatively evaluate changes in airway reactivity; the results showed that only the R animals developed an increase in airway responsiveness (*P < 0.05). Statistical significance was determined using one-way ANOVA followed by Bonferroni’s *post hoc* test (*P < 0.05). Bars and symbols represent the mean ± SEM (*n* = 6 each group).

Guinea pigs were sensitized with a solution composed of OVA grade II (Sigma, St. Louis, MO, USA) 60 μg/mL and Al(OH)_3_ (J.T. Baker, NJ, USA) 1 mg/mL, diluted in physiological saline solution (PSS; PISA, Jalisco, México). The solution was administered intraperitoneally and subcutaneously at a volume of 0.5 mL each. Control animals underwent PSS identically. An antigen boost was performed 8 days after sensitization by exposing the animals to aerosolized OVA (3 mg/mL) for 5 min using an ultrasonic US-1 Bennett nebulizer (flow rate of 2 mL/min; Multistage Liquid Impinger, Burkard Manufacturing Co., Hertfordshire, UK). From day 15 post-sensitization onward, animals were periodically exposed every 10 days to aerosolized OVA challenges: 1 mg/mL for the first challenge and 0.5 mg/mL for the subsequent challenges, until completing 12 challenges (125 days). The control group was challenged with PSS. The aerosol exposures were performed while the animals were placed inside a barometric plethysmography chamber (**Figure 1A**).

### 2.3 Plethysmography

For each antigenic challenge, guinea pigs were placed in a barometric plethysmography chamber for freely moving animals (Buxco Electronics Inc., NY, USA). Diverse parameters, including pressure fluctuations within the chamber during the respiratory cycle (inspiration–expiration), were recorded with a transducer linked to an amplifier and compared with a reference chamber, as previously described by Hamelmann (27) and Bazán-Perkins (28). To avoid artifacts, the program was calibrated to detect changes greater than 1 mL of air, with a minimum inspiratory time of 0.15 s and a maximum inspiratory time of 3 s. Changes in the pressure in the animal chamber were analyzed with Buxco BioSystem XA v1.1 software, which indirectly calculates airway obstruction after exposure to OVA or PSS using the following formula:


$$Bi=\left(\left(Te-Rt\right)/Rt\right)(PEP/PIP)$$

where Bi, broncho-obstruction index; Te, expiratory time (s); Rt, relaxation time (s); PEP, peak expiratory pressure (cmH_2_O); and PIP, peak inspiratory pressure (cmH_2_O). The Bi value represents the average of Bi measurements recorded over a 5-min interval. The Bi was registered 10 min before the challenge and 30 min after the challenge.

### 2.4 Airway responsiveness

The evaluation of airway reactivity was conducted on day 125 by performing two histamine dose–response curves (29, 30). Increasing concentrations of histamine (Sigma, USA; 0.001–0.32 mg/mL) were nebulized to the animals for 1 min at 15-min intervals. The Bi was recorded before initiating the histamine curve (baseline Bi) and after exposure to each histamine concentration, until reaching a concentration that induced a 200% Bi increase compared with the baseline Bi. This histamine concentration is known as the provocative dose 200 (PD200). An OVA challenge was carried out once the Bi had returned to baseline levels, and 3 h later, a second histamine curve was conducted in the same manner as the first. Control animals adhered to the same protocol; however, a PSS challenge was administered between the histamine response curves.

### 2.5 BAL differential cell count

Animals were deeply anesthetized 12 h after the evaluation of airway reactivity with a dose of sodium pentobarbital (28 mg/kg; Pisa, México). BAL fluid was obtained by carefully exposing and cannulating the trachea. Subsequently, 2 × 5 mL of pre-warmed (37 °C) PSS was introduced and recovered by aspiration. Recovered fluid was centrifuged at 1, 500 rpm for 10 min at 4°C. The supernatant was collected and stored with protease inhibitor (Roche, Germany) at −70 °C. The cells were resuspended in 1 mL of PSS and counted in a Neubauer hemocytometer (Hirschmann, Germany), and the volume was adjusted to obtain a concentration of 1 × 10^6^ cells/mL. Cells were stained using May–Grünwald–Giemsa, Romanowsky, staining for differential cell counting. Differential cell counts were performed on 200 cells per sample under light microscopy (40×).

### 2.6 Immunohistochemistry

A section of the left inferior lobe of the lung was obtained and fixed into 10% formalin-buffered solution. Dehydration of the tissue was performed using increasing concentrations of ethanol (50%, 70%, 96%, and 100%), treated with xylene, and embedded in paraffin. Sections of paraffin-embedded lung were cut at 5 μm thickness using a microtome and placed on slides.

For the immunohistochemistry (IHC) procedures, the tissues were deparaffinized at 55°C for 30 min and rehydrated by reversing the previously described dehydration process. Heat-induced epitope retrieval was carried out to restore tissue antigenicity. For this purpose, the slides were immersed in 10 mM of citrate buffer (pH 6.0, 0.05% Tween-20, Bio-Rad, CA, USA) and heated under gentle boiling conditions for 5 min. The samples were incubated for 2 h at room temperature (RT) with blocking buffer, 137 mM of phosphate-buffered saline (PBS, pH 7.4), 3% bovine serum albumin (BSA; Sigma, USA), and 0.1% Tween-20 to prevent non-specific interactions.

Slides were incubated overnight at 4 °C with monoclonal primary antibody anti-laminin α1 (R&D Systems, MAB4656, USA; dilution 10 μg/mL), anti-laminin α2 (clone 4H8-2, Abcam, UK; dilution 1:100), anti-laminin α3 (clone C3, Invitrogen, USA; dilution 1:50), anti-laminin α4 (clone EPR28287-61, Abcam, UK; dilution 1:50), anti-laminin α5 (clone CL3118, R&D Systems, USA; dilution 1:50), anti-laminin β1 (clone DG10, Abcam, UK; dilution 1:50), anti-laminin β3 (clone CL3112, Abcam, UK; dilution 1:500), anti-laminin γ1 (clone BC17, Abcam, UK; dilution 1:100), anti-laminin γ2 (clone CL2980, Thermo Fisher Scientific, USA; dilution 1:500), or polyclonal anti-laminin γ3 (clone C119566, LSBio, WA, USA; dilution 1:50). Endogenous peroxidase activity was blocked by incubation with PBS containing 3% H2O2 for 15 min at RT. Subsequently, the tissues were incubated with horseradish peroxidase (HRP)-conjugated secondary antibodies anti-rat (HRP, Abcam, UK; dilution 1:1, 000), anti-rabbit (HRP, Abcam, UK; dilution 1:1, 000), or anti-mouse (HRP, R&D Systems, USA; dilution 1:100) for 2 h at RT. Tris-buffered saline–Tween-20 buffer (TBST, pH 7.6, 0.1% Tween-20) was used to wash the slides thoroughly between each step. Finally, 3-amino-9-ethylcarbazole (AEC, Invitrogen, USA) or 3, 3′-diaminobenzidine (DAB, Thermo Fisher Scientific, USA) was used as the chromogenic substrate, producing red or brown reaction products, respectively, allowing the chromogenic visualization of antigen. The tissues were counterstained with hematoxylin (Vector Laboratories, USA) and washed with distilled water.

These and the subsequent primary antibodies against different LNs were selected based on availability and on the sequence homology in LNs between human, mouse, and guinea pig LNs. In addition, an analysis of the immunogen (when available) or full protein sequence was performed using the UniProt Basic Local Alignment Search Tool (BLASTp), revealing homology of the sequences from 82.1% to 92.7% for LN α2, LN α4, LN β1, LN β3, and LN γ1; 71.1% to 79.1% for LN α1, LN α3, LN α5, and LN γ2; and 68% for LN γ3.

### 2.7 Semiquantitative IHC expression analysis

Semiquantitative analysis allows the evaluation of protein expression based on the correlation of the chromogen staining intensity with the protein abundance within the tissue (30, 31) using specialized image analysis software. For the semiquantitative analysis, the open-source software ImageJ Fiji (ImageJ2 version 2.16.0/ImageJ 1.54g; 2023-2024, running in a Java 1.8.0_345, 64-bit environment) was calibrated to quantify average pixel color intensity values based on a grayscale, where black pixels correspond to a default value of 0 and white pixels to a value of 250, respectively. Images of immunohistochemically stained tissues were obtained at 10× and 40× magnifications. The captured images were then converted to grayscale for quantitative analysis, consistent with the previous software calibration. Four chromogen-stained quadrants were selected from four ASM and from IVSM regions in each guinea pig (*n* = 6 per group). In this analysis, the software assigns a pixel intensity value of 250 to unstained areas, whereas counterstaining with hematoxylin and specific chromogen (AEC, DAB) produces variable pixel intensity values (lower than 250) depending on the degree of chromogen coloration. Increased antigen–chromogen development results in darker staining areas; however, in the analysis, the darker areas correspond to lower pixel intensity values. To establish a direct correlation between pixel intensity and chromogen staining intensity, the reciprocal intensity was calculated using the formula *r* = 250 − *y*, where *y* represents the pixel intensity of the stained region of interest (30).

### 2.8 Enzyme-linked immunosorbent assay

The presence of soluble LNs that showed positive staining in tissue was evaluated in BAL fluid supernatants and blood serum by enzyme-linked immunosorbent assay (ELISA). Flat-bottom clear polystyrene 96-well plates (Corning Costar, USA) were coated with BAL fluid or blood serum samples diluted 1:10 in carbonate bicarbonate buffer (100 mM, pH 9.6) and incubated overnight at 4 °C. The plates were blocked with blocking buffer (PBS, 5% BSA, 0.05% Tween-20) for 2 h at RT. Samples were then incubated with primary antibody anti-laminin α1 (clone G-12, Santa Cruz Biotechnology, USA; dilution 10 μg/mL), anti-laminin α2 (clone 4H8-2, Sigma, USA; dilution 10 μg/mL), anti-laminin β2 (clone C4, R&D Systems, USA; dilution 10 μg/mL), anti-laminin β3 (clone 2G10, Thermo Fisher Scientific, USA; dilution 10 μg/mL), anti-laminin γ1 (clone BC17, Abcam, UK; dilution 5 μg/mL), or anti-laminin γ2 (clone E-6, Santa Cruz Biotechnology, USA; dilution 15 μg/mL) for 2 h at RT, followed by incubation with HRP-conjugated secondary antibodies for 1 h in the dark. Washing was performed three times using PBS (0.05% Tween-20), with gentle agitation between each step. The chromogenic reaction was developed by adding o-phenylenediamine dihydrochloride (0.5 mg/mL) prepared in citrate buffer (0.05 M, pH 5.0) supplemented with 0.03% hydrogen peroxide. The reaction was stopped by adding sulfuric acid (2 M), and the optical density was determined at 492 nm using a microplate reader (BioTek, USA).

### 2.9 Statistical analysis

Airway responsiveness to histamine induced by antigen challenge was evaluated using the PD200 ratio, defined as the PD200 value post-OVA challenge divided by the PD200 value before the challenge. Broncho-obstructive response data following antigen challenge were analyzed using two-way ANOVA followed by Tukey’s test. Histamine responsiveness induced by antigen challenge, cell counts, semiquantitative IHC expression in ASM and IVSM, and optical density (OD) from ELISAs were analyzed using one-way ANOVA followed by a Bonferroni multiple-comparison *post hoc* test. Data are presented as mean ± SEM from three independent experiments. A Spearman’s correlation analysis was conducted to evaluate the potential relationship between different LN expressions and Bi values after OVA challenge, histamine response (PD200post/PD200pre-challenge), and serum with inflammatory cells.

## 3 Results

### 3.1 Broncho-obstructive response to antigenic challenge

Sensitized animals were classified as R and NR based on their obstructive response following the OVA challenge. A subset of six animals (*n* = 6) per group was selected for further analysis. R animals exhibited a typical obstructive pattern, defined as an increase of ≥200% in basal Bi, after the OVA challenge (**Figure 1B**), consistent with the criteria used to define a significant airway obstruction (20). In contrast, NR animals exhibited either no increase in Bi or an increase <200% relative to basal levels (**Figure 1B**). The maximal response was assessed after each of the 12 OVA challenges, with the R group consistently showing higher Bi (*P* < 0.05) values than the NR group.

### 3.2 Airway responsiveness

To evaluate the effect of antigen exposure on airway responsiveness, we analyzed the response to histamine in the control, R, and NR groups following OVA challenge. Our result showed that in R animals, antigen (OVA) exposure significantly increased airway responsiveness (antigen-induced hyperresponsiveness) to histamine, reflected by a lower PD200 required to provoke a 200% increase in Bi after the OVA challenge and by the PD200post/PD200pre-OVA challenge with a value <1 (**Figure 1C**, P < 0.05). In contrast, the NR group showed no significant difference between PD200 values before and after the antigen challenge (**Figure 1C**). This response was similar to that observed in the control group, indicating that the NR animals did not exhibit increased airway responsiveness following antigen exposure.

### 3.3 BAL cellular counting

The results of BAL cellular counting indicated an increase in some inflammatory cells in the R and NR groups (**Supplementary Figure 1**). In BAL cell counting, R and NR animals showed an increase in lymphocyte counts (*P* < 0.01) compared to controls. Notably, R animals demonstrated a significant elevation in eosinophil numbers (*P* < 0.01), indicating a pronounced eosinophilic response. In contrast, NR animals exhibited an increase in the number of neutrophils (*P* < 0.05).

### 3.4 LN expression

The expression of LNs was analyzed in ASM and IVSM tissues, as well as in fluids such as BAL and serum. Among the LNs analyzed, LN α3, LN α4, LN α5, LN β1, and LNγ3 did not exhibit detectable staining in tissue samples. Consequently, these isoforms were not further analyzed by ELISA. This lack of detection may reflect tissue-specific expression patterns, low expression levels below the detection threshold, or limitations related to antibody specificity.

### 3.5 LN α1 expression in ASM, IVSM, BAL, and serum

**Figure 2:**
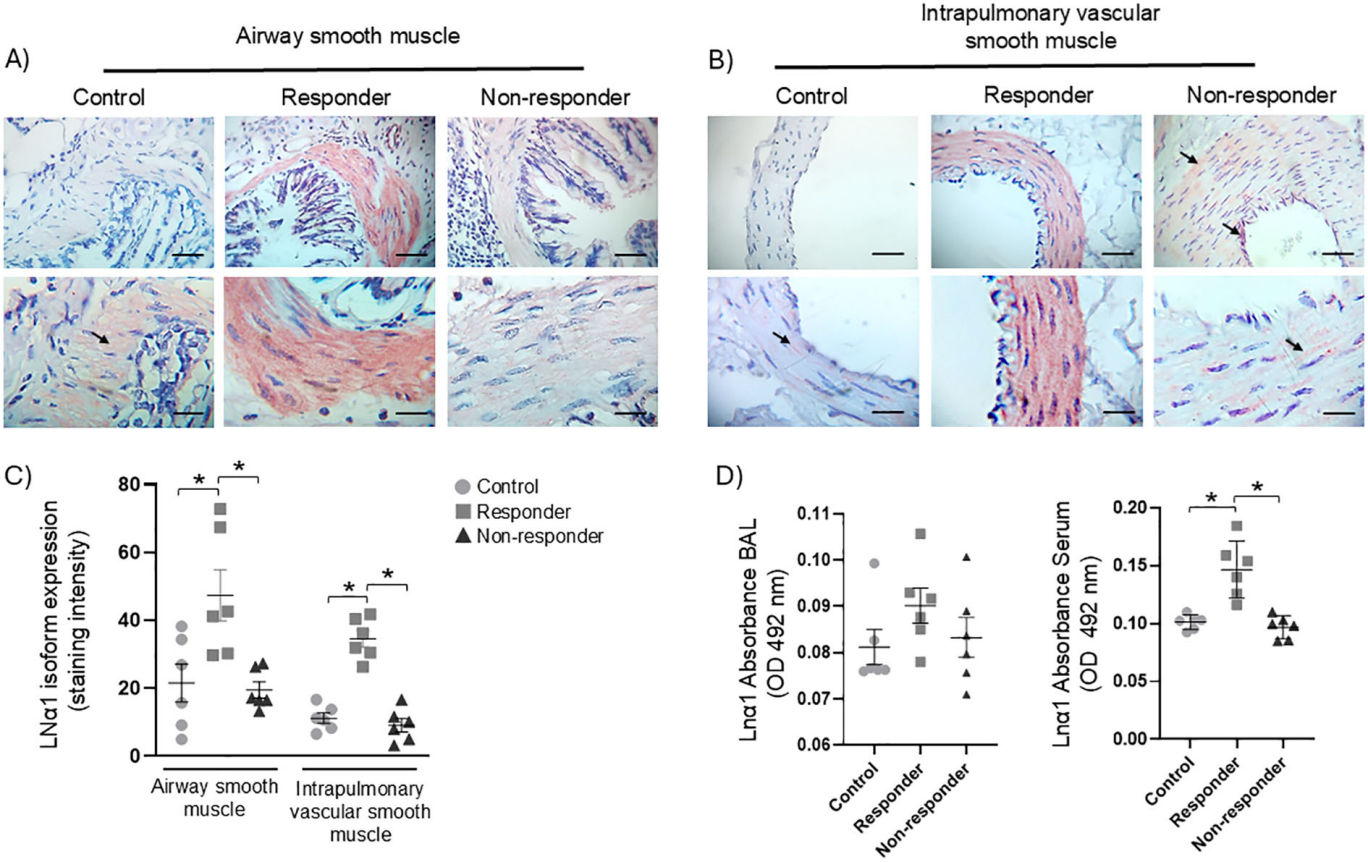
LN α1 isoform expression: **(A, B)** positive immunohistochemical staining (red) was observed in airway smooth muscle (ASM) and intrapulmonary vascular smooth muscle (IVSM) of guinea pigs exhibiting airway obstruction and hyperresponsiveness after antigenic challenges (responder), as well as in those not showing such responses (non-responder), compared to controls. The control and non-responder groups displayed similar staining patterns, with occasional positive bundles (black arrows). Magnifications: 40× and 100×; scale bar: 50 μm; chromogen: AEC. **(C)** Semiquantitative analysis of LN α1 staining intensity in ASM and IVSM. **(D)** Soluble LN α1 levels in BAL and blood serum were measured by ELISA. Statistical significance was determined using one-way ANOVA followed by Bonferroni’s *post hoc* test (*P < 0.05). Bars represent the mean ± SEM (*n* = 6 per group).

LN α1 was expressed in both ASM and IVSM in all groups. The R animals exhibited a more homogeneous distribution of LN α1 (**Figures 2A, B**), along with a significantly increased chromogen staining intensity in ASM and IVSM when compared to the control and NR groups (**Figure 2C**, P < 0.05). No significant differences in staining intensity were detected between the control and NR groups. LN α1 was detected in BAL fluid and in blood serum collected from the three groups; however, no statistically significant differences in LN α1 levels were observed in BAL among the control, R, and NR groups. In contrast, in serum measurements, the R group exhibited a significant increase of LN α1 (**Figure 2D**, P < 0.05).

### 3.6 LN α2 expression in ASM, IVSM, BAL, and serum

**Figure 3:**
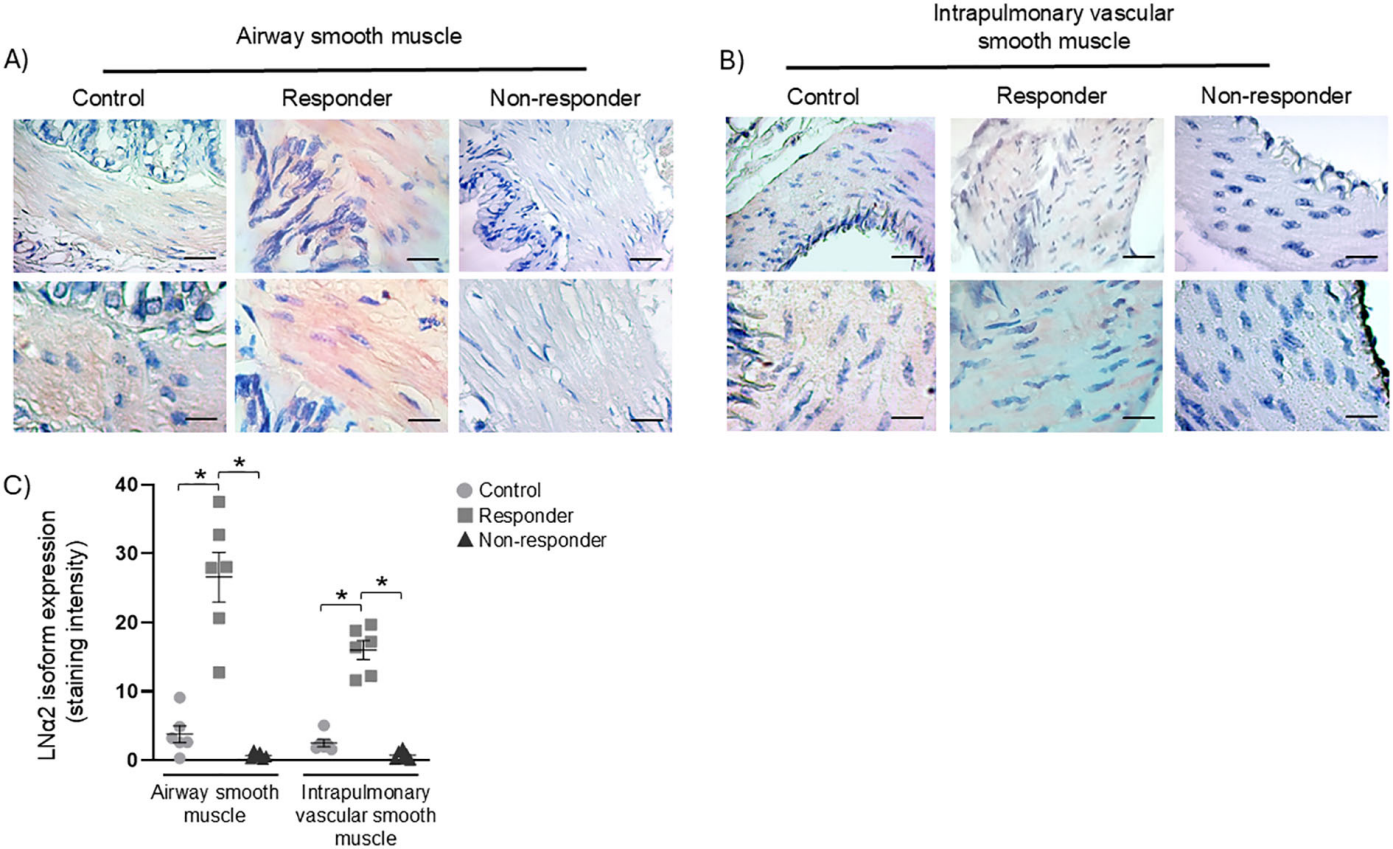
LN α2 isoform expression. **(A)** Immunohistochemical staining for the LN α2 isoform was observed in the airway smooth muscle (ASM) of guinea pigs demonstrating airway obstruction and hyperresponsiveness after antigenic challenges (responders; pink staining), as well as in those without such responses (non-responders), compared to controls. In the control group, a faint staining could be detected in certain regions (black arrows). **(B)** The presence of LN α2 in intrapulmonary vascular smooth muscle appeared as scattered pink bundles (black arrows); this pattern was not observed in the control or non-responder animals. Magnifications: 40× and 100×; scale bar: 50 μm, chromogen: AEC. **(C)** Semiquantitative analysis of LN α2 staining. Statistical significance was determined using one-way ANOVA followed by Bonferroni’s *post hoc* test (*P < 0.05). Bars represent the mean ± SEM (*n* = 6 per group).

Chromogen staining for LN α2 was observed almost exclusively in R animals. Both the control and R groups exhibited chromogen staining in ASM (**Figure 3A**); however, the staining intensity was significantly elevated in the R animals compared with the control and NR groups (**Figure 3C**, P < 0.05). NR animals demonstrated minimal or negligible chromogen staining for LN α2 in ASM. In IVSM, LN α2 expression was visibly lower than that observed in ASM (**Figure 3B**). In R animals, LN α2 was observed in scattered bundles along the muscle which exhibited significantly higher chromogen staining (**Figure 3C**, P < 0.05) compared with the control and NR groups. Control and NR animals displayed few or no detectable chromogen staining. The LN α2 subunit was not detectable in either BAL fluid or blood serum samples.

### 3.7 LN β2 expression in BAL and serum

**Figure 4:**
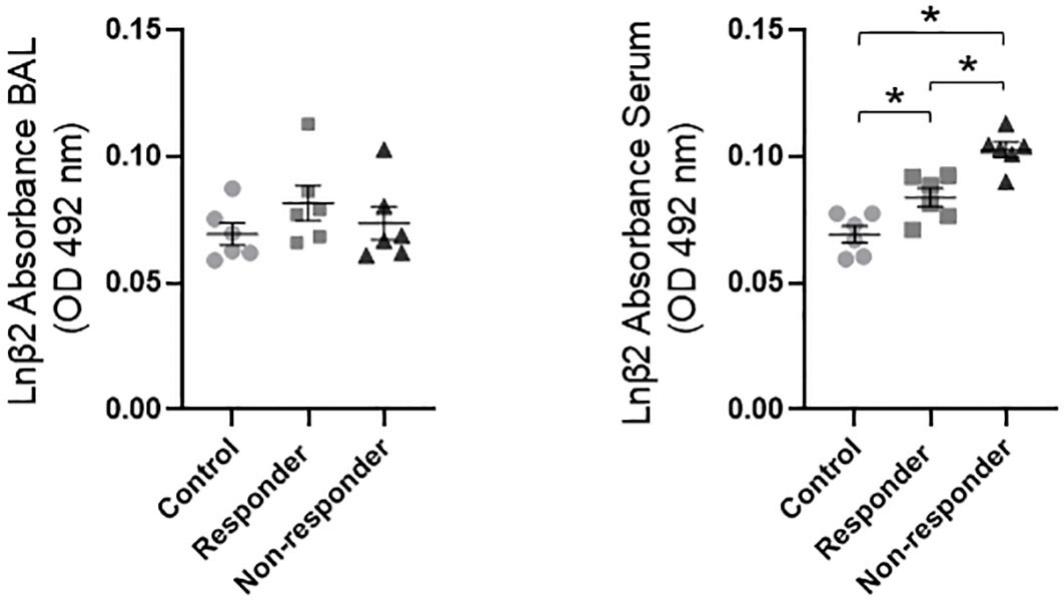
Soluble LN β2 levels in BAL and serum were quantified by ELISA. Statistical significance was evaluated using one-way ANOVA followed by Bonferroni’s *post hoc* test (*P < 0.05). Bars represent the mean ± SEM (*n* = 6 per group).

In a previous study (24), we analyzed the expression of LN β2 in control, R, and NR animals. In that study, an increased expression of LN β2 was found in the ASM and IVSM of the NR group, which was significantly correlated with reduced antigen-induced bronchial obstruction and histamine reactivity. Based on these results, we further investigate LN β2 levels in BAL and serum. This analysis showed a significant (*P* < 0.05) increase in LN β2 levels in the serum of the R group compared to the control group (*P* < 0.05) and in the NR group compared to both the R and control groups (*P* < 0.05; **Figure 4**).

### 3.8 LN β3 expression in ASM, IVSM, BAL, and serum

**Figure 5:**
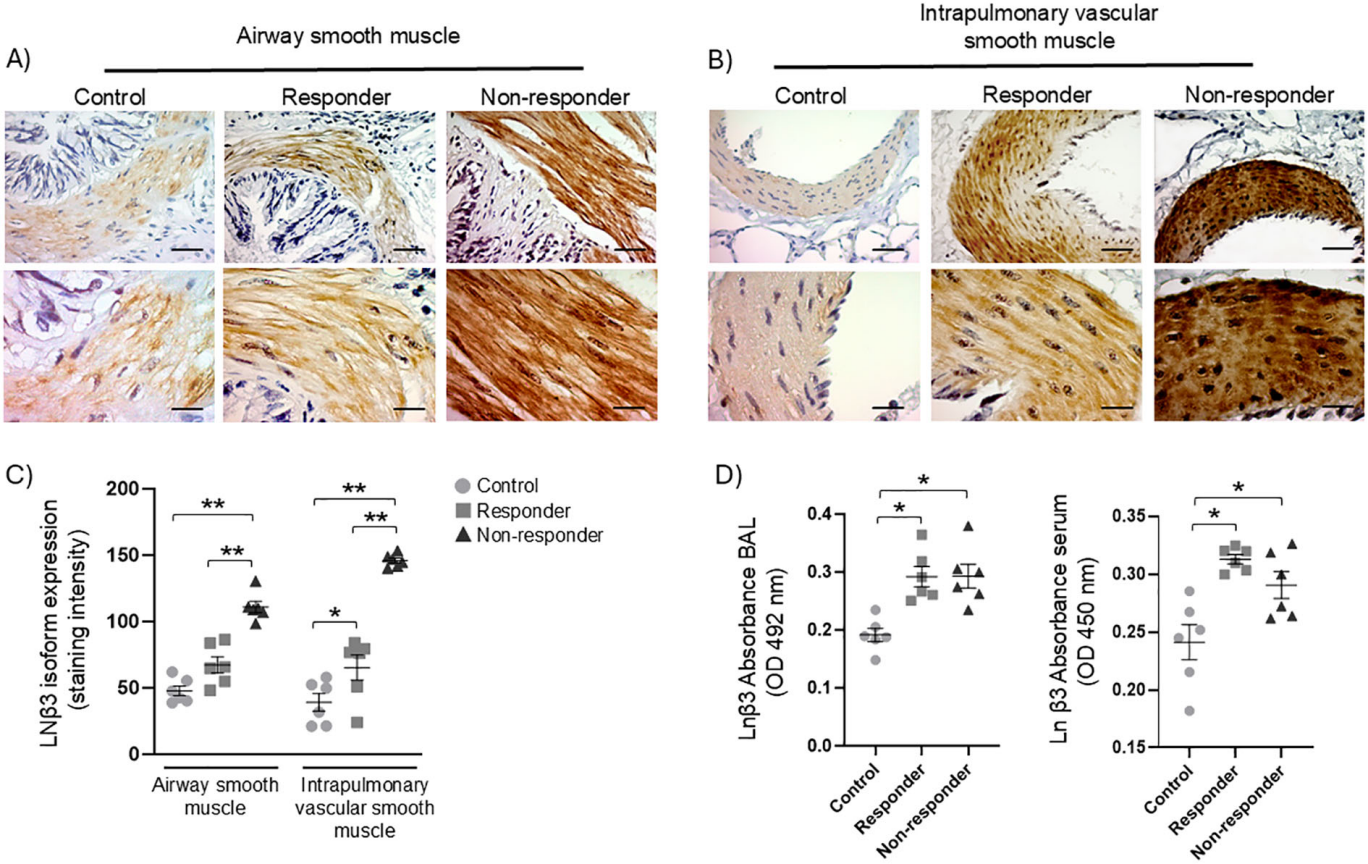
Expression of the LN β3 isoform: **(A, B)** positive immunohistochemical staining (brown) was detected in airway smooth muscle (ASM) and intrapulmonary vascular smooth muscle (IVSM) of guinea pigs demonstrating airway obstruction and hyperresponsiveness after antigenic challenges (responders), as well as in those without such responses (non-responders), compared to controls. Magnifications: 40× and 100×; scale bar: 50 μm, chromogen: DAB. **(C)** Semiquantitative analysis of LN β3 expression in ASM and IVSM. **(D)** Soluble LN β3 isoform in BAL fluid and serum by ELISA. Statistical significance was determined using one-way ANOVA followed by Bonferroni’s *post hoc* test (*P < 0.05; **P < 0.01). Bars represent the mean ± SEM (*n* = 6).

The analysis of the LN β3 showed a wide expression of this subunit in the control, R, and NR groups in ASM and IVSM (**Figures 5A, B**). Quantification of chromogenic staining intensity indicated that, within ASM, NR animals exhibited a significantly higher LN β3 staining intensity compared to both the control and R groups (**Figure 5C**, P < 0.01). In contrast, there was no statistically significant difference in LN β3 staining intensity between the control and R animals.

In IVSM, LN β3 staining intensity revealed notable differences among the three groups (**Figure 5B**). The expression of LN β3 significantly increased in the R group compared to the control (**Figure 5C**, P < 0.05). Notably, NR animals exhibited the highest LN β3 staining intensity compared to control and R animals (**Figure 5C**, P < 0.01). In BAL fluid and in blood serum, the presence of LN β3 subunit was increased in both R and NR animals in comparison with controls (**Figure 5D**, P < 0.05). No significant differences were found between the R and NR groups.

### 3.9 LN γ1 expression in ASM, IVSM, BAL, and serum

**Figure 6:**
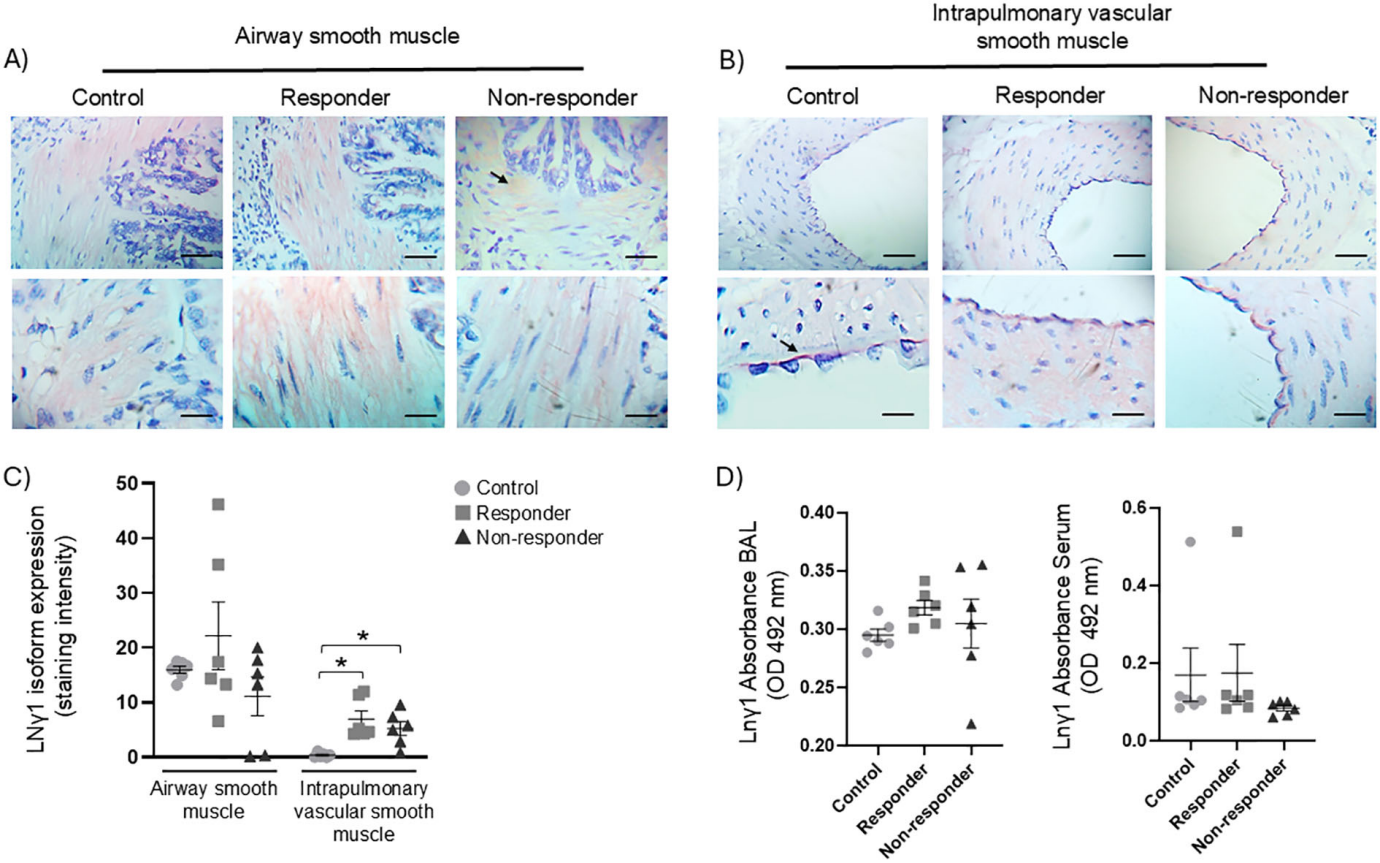
LN γ1 isoform expression: **(A, B)** immunohistochemical staining for the LN γ1 (pink staining) in airway smooth muscle (ASM) of guinea pigs demonstrating airway obstruction and hyperresponsiveness after antigenic challenges (responders), as well as in those without such responses (non-responders), compared to controls. In the intrapulmonary vascular smooth muscle (IVSM) of the control group, LN γ1 expression was restricted to the endothelium of the vascular structure. In contrast, in responder and non-responder animals, the staining extended into the smooth muscle layer (pink staining and black arrows). **(C)** Semiquantitative analysis of LN γ1 expression in ASM and IVSM. **(D)** Soluble LN γ1 isoform in BAL and serum by ELISA. Statistical significance was determined using one-way ANOVA followed by Bonferroni’s *post hoc* test (*P < 0.05). Bars represent the mean ± SEM (*n* = 6 per group).

LN γ1 was expressed in ASM in all three groups with no significant difference in intensity staining (**Figures 6A, C**). In IVSM, the distribution of LN γ1 differed between groups (**Figure 6B**). In the control group, LN γ1 location was limited to the BM in the endothelium, whereas in R and NR, this subunit was expressed in the IVSM, accompanied by a notable increase in staining intensity (**Figure 6C**, P < 0.05). When evaluating BAL fluid and blood serum samples, LN γ1 levels did not show statistically significant differences among the control, R, and NR groups (**Figure 6D**).

### 3.10 LN γ2 expression in ASM, IVSM, BAL, and serum

The expression of LN γ2 was observed in ASM and IVSM in all the groups; nevertheless, the analysis of chromogen staining intensity revealed no significant differences in any tissue (**Supplementary Figures 2A–C**). The presence in BAL and serum did not differ in any group (**Supplementary Figure 2D**).

### 3.11 LN correlations with pathophysiological and inflammatory patterns

Elevated LN α1 and LN α2 expression in ASM showed a significant correlation with increased antigen-induced bronchial obstruction after OVA challenge and reactivity to histamine. In contrast, the expression of LN β3 and LN γ1 did not show a significant correlation with either bronchial or histamine reactivity (**Table 1**).

**Table 1:** Correlation between LN expression in ASM with airway obstruction and responsiveness after OVA challenge.

	Bi value after OVA challenge		PD_200_post/PD_200_pre-challenge	
Laminin isoform (LN)	Correlation coefficient (*r*)	*P-*value	Correlation coefficient (*r*)	*P-*value
LN α1	**0.65**	**0.003**	**−0.58**	**0.01**
LN α2	**0.61**	**0.006**	**−0.61**	**0.006**
LN β3	−0.04	0.87	0.095	0.7
LN γ1	−0.09	0.7	0.25	0.31

Bold values indicate statistically significant correlations.

The analysis of the relationship between LN expression in BAL fluid and the inflammatory cells present in this fluid revealed that the increase of LN β3 in BAL was associated with an increase in lymphocytes, eosinophils, and neutrophils. Additionally, LN γ1 in this fluid showed a correlation with the number of eosinophils in BAL (**Table 2**).

**Table 2:** Correlation between LN expression and inflammatory cell counts in BAL.

	Lymphocytes		Eosinophils		Neutrophils	
Laminin isoform (LN)	Correlation coefficient (*r*)	*P-*value	Correlation coefficient (*r*)	*P-*value	Correlation coefficient (*r*)	*P-*value
LN α1	0.17	0.48	0.19	0.43	0.027	0.91
LN β2	0.38	0.11	0.31	0.2	0.36	0.13
LN β3	**0.52**	**0.02**	**0.64**	**0.003**	**0.645**	**0.003**
LN γ1	0.34	0.16	**0.53**	**0.02**	0.21	0.38

Bold values indicate statistically significant correlations.

## 4 Discussion

The different physiological responses in the R and NR groups were accompanied by differential LN expression, particularly in ASM and IVSM. Immunohistochemical analyses indicated that LN immunoreactivity was detected in ASM and IVSM tissues and was not restricted to the BM or surrounding myocytes but instead displayed a broader distribution throughout the ASM and IVSM tissues. This distribution may contribute to the observed difference in physiological responses, since laminin accumulation in smooth muscle has been associated with functional changes through alterations in its mechanical properties (9, 15).

LN α1 and LN α2 were highly expressed in the ASM and IVSM of R animals. These LNs have been shown to play a role in asthma and contraction remodeling. For instance, LN α1 is essential and almost exclusively involved in the lung development stage (32–34) and low expression in the lungs of adult mice (35); therefore, its overexpression in adult ASM has been associated with remodeling processes (34–39), in which it could perform functions analogous to those observed during development such as maintaining the organization of ASM (35, 40, 41). Furthermore, LN α1 has been reported in patients diagnosed with different types of asthma, in subepithelial BM (36–38) and ASM (38). On the other hand, LN α2 has been associated with the differentiation of mesenchymal cells into myocytes and with the promotion of smooth muscle cells’ survival by the accumulation of anti-apoptotic proteins (41–43). This LN is essential for ASM contraction, since sensitized Lama2^−^/^−^ mice showed the absence of broncho-obstructive responses after antigenic challenge and lacked airway hyperresponsiveness (42, 43). Remarkably, our results demonstrated that both LN α1 and LN α2 were correlated with the increased antigen-induced bronchial obstruction and increased reactivity to histamine. These results align with the reported function of LN α2 in promoting ASM contraction. While LN α2 is linked to contraction, LN α1 may be involved in modifications of BM as a remodeling process within airways. Our results also suggest a potential role in airway contraction for its expression in animals with obstruction and histamine responsiveness.

As in ASM, the presence of LN α1 in IVSM has not been reported in healthy conditions (32, 44). In contrast, LN α2 has been reported in other IVSM (44). The expression of LN α1 and LN α2 in IVSM has been scarcely investigated. Nonetheless, studies have documented alterations in the extracellular matrix and BM in remodeled and proliferative vascular structures in patients with asthma (45, 46). The expression of these LNs in ASM and IVSM of the R group suggests a possible involvement of these isoforms in vascular changes and remodeling processes, although further research is required to elucidate their specific implications.

Interestingly, no increase in LN α1 and LN α2 expression was observed in NR animals, which showed no broncho-obstructive response or histamine responsiveness, but instead exhibited a marked expression of LN β3 in ASM and IVSM. In general, LN β3 has received limited research attention due to the absence of significant changes in its expression and its non-essential role in lung development (47, 48). However, the expression of LN β3 in the adult lung has been attributed to repair processes in response to damage or in pulmonary diseases such as asthma or tumors (47, 48).

In this study, we found that the control group expressed LN β3, indicating that it is an LN expressed in normal conditions. These results agree with the data in which LN β3 mRNA was found in the lungs of healthy adult mice (35), suggesting that LN β3 expression is not limited to damage. Nonetheless, an overexpression of LN β3 was observed in the NR group. This increase was not coordinated with LN γ2, which was similarly expressed in the three groups, an LN that forms a trimer with LN β3 in laminin 332. Laminin 332 is typically found in hemidesmosome junctions, structures known for establishing strong attachments that provide resistance to mechanical stress (49, 50); particularly, LN β3 is known to participate in extracellular matrix assembly, regulating cell adhesion through interactions with matrix proteins such as collagen VII (49–51). These data suggest that the overexpression of LN β3 may be associated with increasing stiffness of ASM in NR animals, generating a hypocontractile phenotype in this group similar to what has been reported for LN β2 (24). However, analysis of the correlation of LN β3 and antigen-induced bronchial obstruction and reactivity to histamine did not show a significant correlation, suggesting that this isoform may not be directly involved in the mechanisms driving airway contraction.

Analysis of LN γ1 expression showed a consistent pattern in ASM in the control, R, and NR groups, reflecting a stable expression in all the groups. In contrast, LN γ1 in vascular structures exhibits differences in its location. In the control group, LN γ1 expression is limited to the BM of the endothelium, indicating a restricted distribution in basal conditions. However, in R and NR animals, LN γ1 expression extends beyond the endothelium, being detected in IVSM, reflecting that antigen exposure can alter LN γ1 expression. The implications of this alteration are unknown, but diverse studies have demonstrated that LN γ1 is essential to maintain vascular smooth muscle survival (43, 52).

Overexpression of BM components is a characteristic of inflammatory lung diseases. LN deposition has been reported in remodeled airways of patients with asthma and COPD. This accumulation may influence cells, including ASM, since some studies have highlighted the importance of ASM–LN interactions during remodeling processes, affecting survival, contraction, and inflammation (41, 42, 53). For example, in a guinea pig model, the administration of the peptide YIGSR, which mimics LN β1, can increase ASM contractility in animals challenged with saline solution as well as in those exposed to allergen; notably, in allergen-challenged animals, YIGSR further increased airway fibrosis and eosinophilic inflammation (53). These findings indicate that the interaction between ASM and laminin plays a critical role not only in contraction but also in remodeling and inflammation. In this context, our findings suggest that chronic inflammation is associated with LN overexpression and that different LNs may have different effects on ASM; in this case, LN α1 and LN α2 appear to be associated with a contractile phenotype, whereas LN β3 and LNγ1 are not. These observations provide a basis for mechanistic studies to elucidate the specific ASM–LN interactions during airway remodeling.

Some of these LNs were also found in soluble form. The detection of soluble laminin in BAL and blood serum has been documented in various lung diseases; this presence serves as an indicator of alterations in the BM, suggesting disruptions in BM integrity that can lead to the release or shedding of LN to the extracellular medium (6, 18, 54, 55). In this study, the analysis of soluble LN revealed an increase in LN α1 in soluble form in the blood serum of R animals, whereas LN β2 and LN β3 were increased in BAL and blood serum of the R and NR groups. This finding suggests that the release of LN β3 into extracellular fluids may be a common feature in these models, regardless of their airway responsiveness status, whereas LN α1 is exclusively increased in the R model. Whether these soluble LNs have a functional role in the extracellular milieu remains unclear, and further studies are needed to clarify their potential involvement. However, our results showed a distinct relationship between the expression of specific LN in BAL and serum and the presence of inflammatory cells. LN α1 in serum, which was increased in the R group, and LN γ1 in serum were found to correlate with an increase in eosinophil numbers. Additionally, LN β3, which increased in R and NR animals, is correlated with the increases in lymphocytes and eosinophils in BAL and serum and with neutrophils in BAL, suggesting that different LNs may be associated with promoting lymphocyte, eosinophil, and neutrophilic inflammation. However, the precise mechanism underlying these possible associations remains unclear.

Sensitized R and NR animals develop inflammation, with an increase in lymphocytes in both groups, eosinophil predominance in the R group, and neutrophils in the NR group. During chronic inflammation, BM can be disrupted; in this context, the increase in LN α1 and LN γ1 in BAL with an increase of eosinophils and LN β3 in BAL and serum is correlated with the number of lymphocytes and eosinophils and with neutrophils in BAL. Interestingly, although the NR group does not show airway obstruction, they present increased neutrophil counts and exhibit changes in LNs, indicating that inflammation and structural alterations can occur even in the absence of broncho-obstruction. These findings also highlight the complexity of the possible involvement of LNs in airway broncho-obstructive and immunological responses, pointing to the possibility that LNs may influence the behavior of ASM and IVSM and may be involved with specific inflammatory cells.

The present study has two considerations. First, it focused exclusively on male guinea pigs. It has been reported that sexual hormones are linked to different protein expression in ASM. For instance, in rats, sexual hormones regulate the expression of estrogen, progesterone, and androgen receptors in ASM, highlighting that hormones can influence smooth muscle biology (56). Therefore, potential sex-related differences in LN expression and airway responses cannot be excluded and warrant further investigation. In addition, this study relied on antibody-based detection in guinea pig tissues. The antibodies were selected based on sequence homology; nevertheless, we cannot exclude the possibility that LNs not detected in this study may also be present in guinea pig airways.

One important consideration is the potential translational relevance of these findings to human asthma. Guinea pig models represent valuable and well-established resources for the study of the mechanisms underlying human respiratory diseases (23). In this study, R and NR guinea pigs appear to represent distinct allergic physiological responses to antigen exposure, with R animals resembling a classic allergic asthma phenotype, while NR animals display features comparable to a putative asymptomatic or subclinical condition. The differential overexpression of specific LNs observed in each group may reflect distinct mechanisms contributing to airway remodeling and altered ASM and IVSM mechanics, phenomena that are well described in asthmatic patients. Notably, the findings in NR animals suggest that structural alterations and inflammation can occur independently of overt bronchoconstriction, a scenario that may be relevant to asymptomatic or underdiagnosed conditions in humans. Although further validation in human tissues is required, these results support the concept that specific LNs may differentially modulate ASM and IVSM behavior and could potentially serve as biomarkers or therapeutic targets associated with airway remodeling in allergic airway diseases.

**Figure 7:**
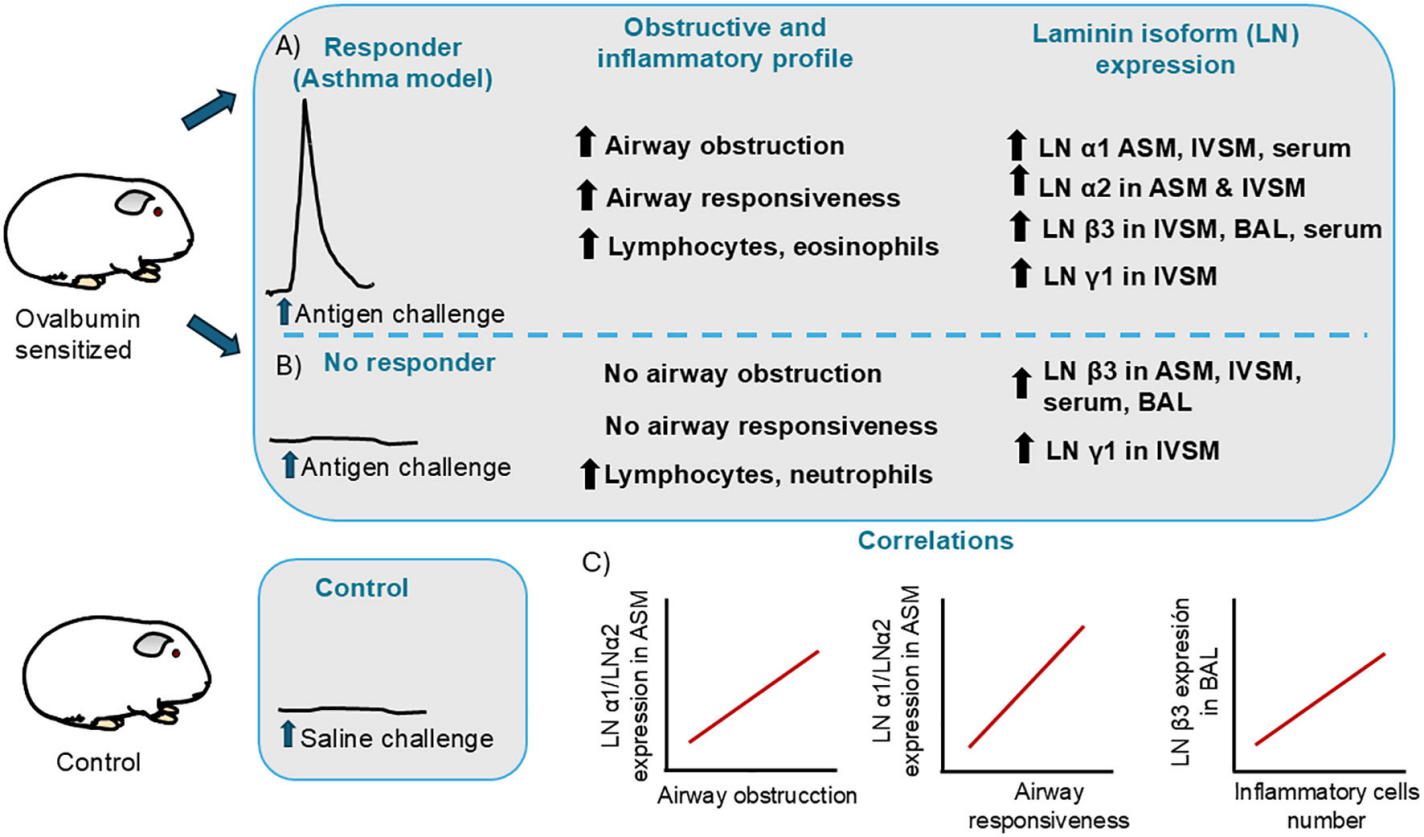
Laminin isoform (LN) expression in sensitized guinea pigs. OVA-sensitized guinea pigs develop two different obstructive phenotypes after antigen challenge: responders (R) and non-responders (NR). **(A)** R animals (asthma model) consistently show an increase in airway obstruction after challenge, airway hyperresponsiveness, and inflammation characterized by lymphocytes and eosinophilic presence in bronchoalveolar lavage (BAL). After chronic antigen challenge, R animals exhibit increased expression of LN α1 and LN α2 in airway smooth muscle (ASM), while intrapulmonary vascular smooth muscle (IVSM) shows increased expression of LN β3 and LN γ1. Some LNs were detected in soluble form. In R animals, the levels of LN α1 were increased in serum and those of LN β3 were increased in serum and BAL. **(B)** The NR guinea pigs do not develop increased airway obstruction or airway hyperresponsiveness after antigen challenge. These animals show increased lymphocytes and neutrophils in BAL. In NR animals, overexpression of LN β3 is observed in both ASM and IVSM, and LN γ1 is overexpressed in IVSM. LN β3 was detected increasing in NR serum and BAL. **(C)** The increase of LN α1 and LN α2 in ASM was correlated with airway obstruction and airway hyperreactivity, whereas the increase of soluble LN β3 was correlated with the presence of inflammatory cells.

In conclusion, R and NR are two sensitized animal groups that differ not only in their physiological responses to antigen exposure but also in their laminin expression patterns, which may underlie these differences (**Figure 7**). LN α1 and LN α2 are overexpressed in the R group, and these LNs are associated with an increase in antigen-induced bronchial obstruction and a high responsiveness to histamine. Although the expression of these LNs in IVSM has been less explored, it may reflect vascular alterations in this group. In contrast, the NR group shows overexpression of LN β3, which does not appear to be directly involved in the mechanism driving airway contractile or hypocontractile phenotype.

## Data Availability

The original contributions presented in the study are included in the article/**Supplementary Material**. Further inquiries can be directed to the corresponding authors.
