# Supplementary material for: Analyzing soluble and lung smooth muscle laminin isoform expression in guinea pigs exhibiting various allergic response phenotypes
Source: Front Immunol. 2026 Mar 24;17:1774996. doi: 10.3389/fimmu.2026.1774996 (PMC13053283; doi:10.3389/fimmu.2026.1774996)
Supplement: Supplementary file 1 [file DataSheet1.docx]

Supplementary Material

# Supplementary Data

Bronchoalveolar lavage (BAL) cell counts were performed to assess inflammatory cell profiles in control, responder (R) and non-responder (NR) guinea pigs. Total cells per group are shown in Supplementary Figure S1. Lymphocyte numbers were significantly elevated in R and NR animals (P < 0.01). In addition, R animals showed a marked increase in eosinophil counts (P < 0.01), reflecting a predominant eosinophilic inflammatory profile. Conversely, NR animals presented a significant rise in neutrophil numbers (P < 0.05).

The LN γ2 expression present in airway smooth muscle (ASM) and in intrapulmonary vascular smooth muscle (IVSM) in control, R and NR groups showed similar staining distribution and intensity in both muscles along the three groups of animals (Supplementary Figures S2A-C). In addition, LN γ2 levels in BAL fluid and serum were comparable among groups (Supplementary Figure S2D).

#
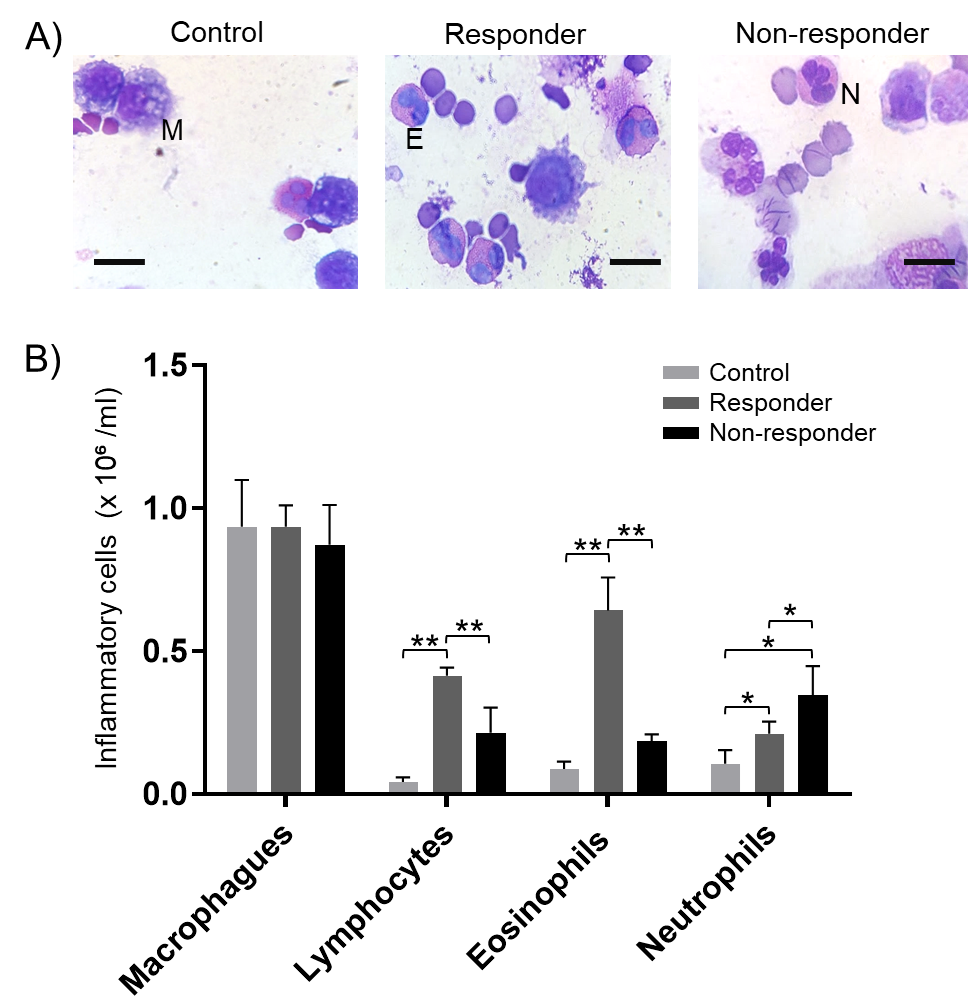
Supplementary Figures

**Supplementary Figure 1.** Inflammatory cells in bronchoalveolar lavage (BAL). (A) Representative cells in BAL of guinea pigs with (responders) or without (non- responders) airway obstruction obstruction after antigenic challenge, and control animals. M: Macrophage, E: Eosinophil, N: Neutrophil. Magnification: 40x; scale bar: 20 μM. (B) Cell counting in BAL of control, responder and non-responder guinea pigs. Statistical significance was determined using one-way ANOVA followed by Bonferroni’s post hoc test (*P < 0.05, **P<0.01). Bars represent the mean ± SEM (n = 6).


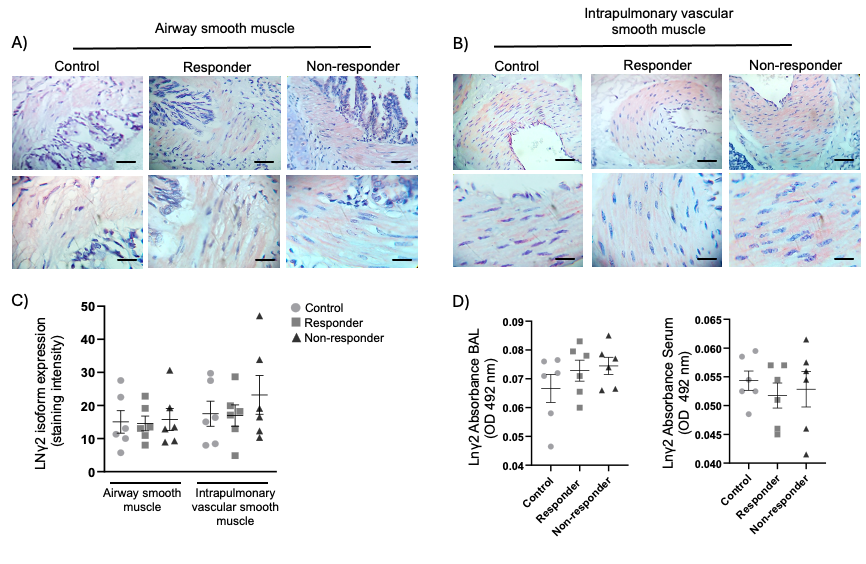


**Supplementary Figure 2.** LNγ2 expression. (A) and (B) Positive immunohistochemical staining (light red) was observed in airway smooth muscle (ASM) and intrapulmonary vascular smooth muscle (IVSM) of guinea pigs with (responders) and no (non- responders) airway obstruction after antigenic challenge and in controls. Magnification: 40x and 100x; scale bar: 50 μM; chromogen: AEC. (C) Semi-quantitative analysis showed a similar staining intensity displayed in the three groups. (D) Soluble LNγ2 levels in BAL and serum were measured by ELISA. Statistical significance was determined using one-way ANOVA followed by Bonferroni’s post hoc test (*P < 0.05). Bars represent the mean ± SEM (n = 6).
